# Comparative transcriptomic analysis reveals key components controlling spathe color in *Anthurium andraeanum* (Hort.)

**DOI:** 10.1371/journal.pone.0261364

**Published:** 2021-12-10

**Authors:** Jaime A. Osorio-Guarín, David Gopaulchan, Corey Quanckenbush, Adrian M. Lennon, Pathmanathan Umaharan, Omar E. Cornejo

**Affiliations:** 1 Centro de Investigación Tibaitatá, Corporación Colombiana de Investigación Agropecuaria–Agrosavia, Mosquera, Cundinamarca, Colombia; 2 Faculty of Science and Technology, Department of Life Sciences, The University of the West Indies, St. Augustine, Republic of Trinidad and Tobago; 3 Division of Molecular and Translational Sciences, U. S. Army Medical Research Institute of Infectious Diseases (USAMRIID), Fort Detrick, MD, United States of America; 4 School of Biological Sciences, Washington State University, Pullman, Washington, United States of America; Youngstown State University, UNITED STATES

## Abstract

*Anthurium andraeanum* (Hort.) is an important ornamental in the tropical cut-flower industry. However, there is currently insufficient information to establish a clear connection between the genetic model(s) proposed and the putative genes involved in the differentiation between colors. In this study, 18 cDNA libraries related to the spathe color and developmental stages of *A*. *andraeanum* were characterized by transcriptome sequencing (RNA-seq). For the *de novo* transcriptome, a total of 114,334,082 primary sequence reads were obtained from the Illumina sequencer and were assembled into 151,652 unigenes. Approximately 58,476 transcripts were generated and used for comparative transcriptome analysis between three cultivars that differ in spathe color (‘Sasha’ (white), ‘Honduras’ (red), and ‘Rapido’ (purple)). A large number of differentially expressed genes (8,324), potentially involved in multiple biological and metabolic pathways, were identified, including genes in the flavonoid and anthocyanin biosynthetic pathways. Our results showed that the chalcone isomerase (*CHI*) gene presented the strongest evidence for an association with differences in color and the highest correlation with other key genes (flavanone 3-hydroxylase (*F3H*), flavonoid 3’5’ hydroxylase (*F3’5’H)/* flavonoid 3’-hydroxylase (*F3’H)*, and leucoanthocyanidin dioxygenase (*LDOX*)) in the anthocyanin pathway. We also identified a differentially expressed cytochrome *P450* gene in the late developmental stage of the purple spathe that appeared to determine the difference between the red- and purple-colored spathes. Furthermore, transcription factors related to putative MYB-domain protein that may control anthocyanin pathway were identified through a weighted gene co-expression network analysis (WGCNA). The results provided basic sequence information for future research on spathe color, which have important implications for this ornamental breeding strategies.

## Introduction

The large diversity of floral traits in angiosperms have fascinated researchers and ornamental flower enthusiasts alike for generations. Evolutionary biologists have long held that the large diversity of floral traits is associated with high rates of diversification among species, a process that is likely mediated by natural selection [1]. Among floral traits, flower color and scent are undoubtedly of great evolutionary importance given the direct impact they can have on pollinator choice and the plant’s fitness [2–4].

*Anthurium andraeanum* (Hort.) is a species complex created by interspecific hybridization between *A*. *andraeanum* Linden ex André and other related species [5]. The large diversity in spathe colors such as white, red, pink, orange, coral, green, brown, and purple and patterns such as obake, striped, and colored borders [5–7] has made this species an important tropical ornamental crop [8]. It is well known that anthocyanins (synthesized in the cytosol and localized in vacuoles), widely found in the flowers, seeds, fruits, and vegetative tissues of vascular plants as soluble flavonoid, confers pigmentation and also participate in the defense against a variety of biotic and abiotic stressors in plants [9–11]. Besides being directly beneficial to plants, anthocyanins have also been used as natural food colorants and display vital nutraceutical properties that could be advantageous for human health [11–13].

Extensive crossing analyses have been done on cultivars of *Anthurium* to unveil the genetic mechanisms involved in the color formation and segregation [6,14]. Elibox and Umaharan (2008) showed that spathe color is controlled by duplicate recessive epistasis and proposed two genes (O and R) as responsible for the distinction between color vs white and a modifier gene (M) as responsible for the distinction between reds and pinks/oranges [14]. The biosynthetic pathway generating anthocyanins is highly conserved in plants and the genes involve has been extensively studied [15]. For example, the chalcone synthase (*CHS*) gene catalyzes the first reaction leading to anthocyanin biosynthesis and assists in forming the intermediate chalcone, which is the primary precursor for all classes of flavonoids. The chalcone isomerase (*CHI*) gene, is the second key enzyme in the anthocyanin biosynthetic pathway that catalyzes the stereospecific and intramolecular isomerization of naringenin chalcone into its corresponding (2*S*)-flavanones. Dihydroflavonol 4-reductase (*DFR*) gene is the first committed of anthocyanin biosynthesis in the flavonoid biosynthetic pathway and is responsible for the formation of leucoanthocyanidins which can be converted into colored anthocyanidins by the anthocyanidin synthase (*ANS*) gene [11]. The enzymes which catalyze specific steps of the anthocyanin biosynthesis pathway are encoded by structural genes which are in turn under the control of regulatory genes (transcription factors (TF)). For example, TFs like R2R3-MYB and members of the basic helix-loop-helix (bHLH) (R/B) family, have been reported as regulatory elements that controls pigmentation in flowers and fruits in different species [16–20]. A putative TF AaMYB2, ectopically expressed in tobacco increased anthocyanin accumulation and increased the expression of *DFR*, flavonoid 3’-hydroxylase (*F3’H*), *ANS*, and possibly *CHS* genes [21]. However, regulatory genes driving the expression of the enzymes in the pathway might differ depending on the species [18–26].

In *A*. *andraeanum*, the major color pigments in the spathe are anthocyanins, predominantly cyanidin, and pelargonidin derivatives, of which the content and ratio determine the color and its intensity [27]. A study showed that the interaction between a R2R3-MYB TF with a basic helix-loop-helix (AabHLH1) regulates the proanthocyanidin accumulation [28]. Besides, temporal and spatial expression analysis of genes involved in flavonoid synthesis (*CHS*, *F3’H*, *F3H* (Flavanone 3-hydroxylase) *DFR*, and *ANS*) suggests that *DFR* transcript levels vary significantly between developmental stages and might represent a relevant regulator [29]. A phenotype and transcriptome analysis of anthurium leaf color mutants suggested that color formation in leaves was greatly affected by chloroplast development and pigment biosynthesis with increased expression of flavonoid 3’5’ hydroxylase (*F3’5’H*) and *DFR* genes in the dark green leaf mutant compared to the wildtype [30]. However, the genes involved in the regulation of color in these mutants could not be identified. Thus, a more detailed analysis of gene expression was essential to improve our understanding between the genetic model(s) and the putative regulatory genes involved in the differentiation between colors. Prior studies have focused on the analysis of specific genes within the pathway [29,30], sometimes failing to perform appropriate comparisons between white cultivars and colored ones or providing quantitative support for the observed differences [29].

Approaches such as RNA sequencing (RNA-Seq) have allowed the profiling of global gene expression patterns and the mapping of simple or complex traits, respectively, both in model and non-model plants. Several recent studies have exploited this technology to study traits of particular interest in a wide range of species due to reduction in library construction and sequencing costs [23,31–33]. In this work, we performed a comprehensive transcriptomic analysis of white, red, and purple spathes of three cultivars of *A*. *andraeanum* from two cut-flower development stages. The aims of our study were: 1) to generate a new reference transcriptome based on the Honduras cultivar, 2) to identify differentially expressed genes directly involved in the biosynthesis of anthocyanins, and 3) to identify putative regulatory genes involved in the expression of spathe color. The present study provides an important molecular basis for understanding the color formation mechanism for germplasm evaluation and ornamental breeding.

## Materials and methods

### Plant material

**Fig 1:**
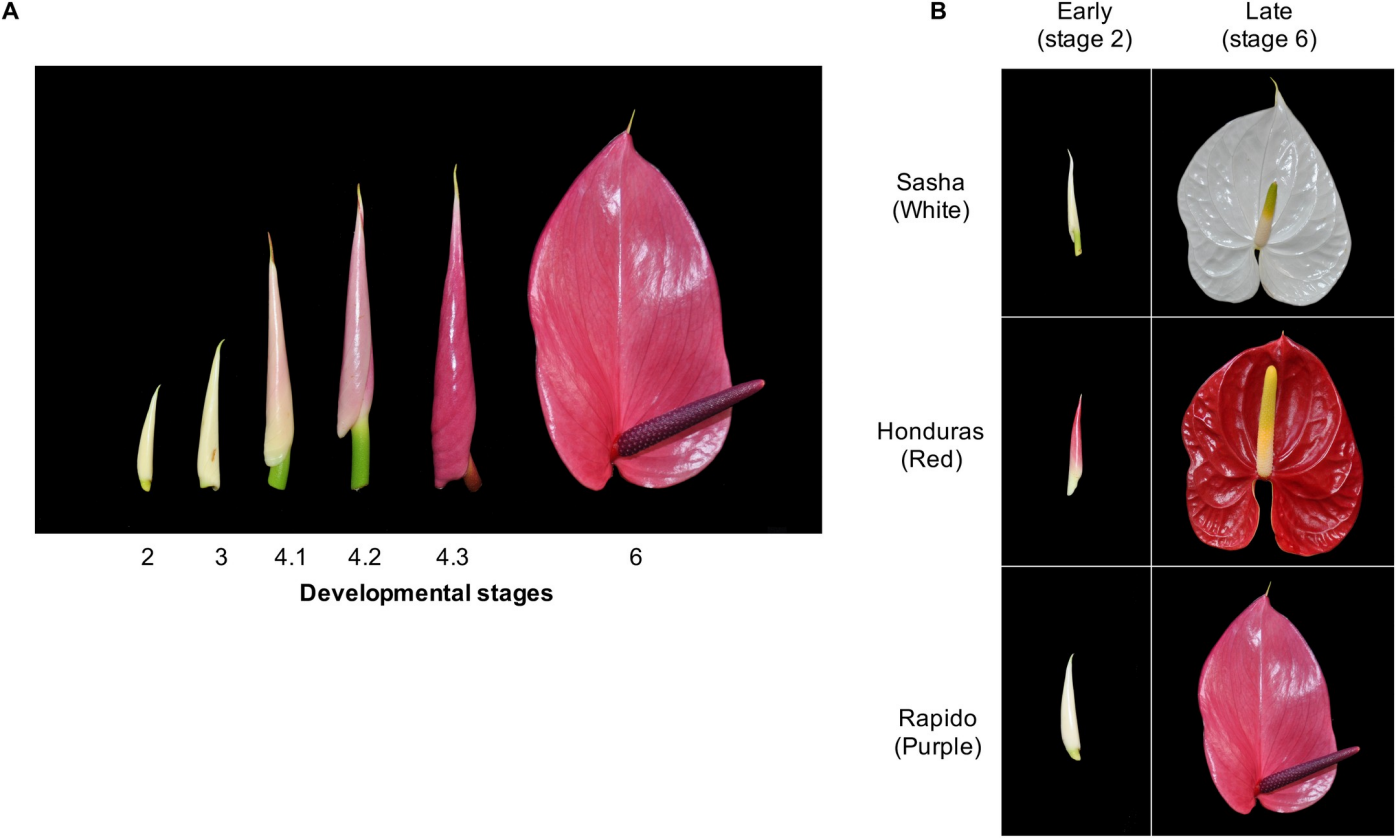
Cultivars and stages of *Anthurium andreaeanum* used in the present study. (A) Stages of spathe development for *Anthurium andraeanum*. (B) Stages 2 and 6 used for each of the three cultivars for the RNA-seq assay.

To identify the genes differentially expressed between white (W), red (R), and purple (P) spathes, cut-flowers were collected from mature 3- to 4-year-old plants of ‘Sasha’, ‘Honduras’, and ‘Rapido’ cultivars respectively (Fig 1). The samples were obtained from Kairi Blooms Ltd., a commercial anthurium farm located at Carapo village, Trinidad, and were harvested in triplicate (3 individual spathes from different plants) from two development stages: early (E) (stage 2 -cut-flower first visible) and late (L) (stage 6-spathe newly opened and fully expanded) (Fig 1). All samples were harvested and immediately stored in liquid nitrogen.

### RNA isolation, library preparation and, sequencing

RNAqueous Kit (Applied Biosystems, Foster City, CA) was used for total RNA isolation from spathe samples of three replicates from each of three cultivars and two developmental stages following the manufacturer’s guidelines. DNA was removed from the samples using Turbo DNA-free (Applied Biosystems, Foster City, CA), also according to the manufacturer’s instructions. Samples were then shipped in dried ice to Washington State University where they were purified with ethanol (70%) and resuspended in 25 μL of ultrapure water. The quantity of isolated RNA was checked using a Qubit fluorometer and the quality of each sample was determined on an Agilent 2100 Bioanalyzer.

To construct the RNA libraries, the Illumina TruSeq Stranded Total RNA with Ribo-Zero Plant kits were used according to the manufacturer’s instructions. To assess the quality and determine the molarity of the prepared libraries, each sample was run on an Agilent 2100 Bioanalyzer. The samples were then normalized to 10 nM, randomized, and sequenced in a single lane (for a total of two lanes) of Illumina HiSeq 2500 System (paired-end, 100 bp reads) at the Washington State University Genomics Core. Raw Sequence data for 18 samples of this study are available under the BioProject ID PRJNA721430.

### *De novo* transcriptome of anthurium and functional annotation

Total RNA was extracted as previously described and combined into one pool and shipped to Beijing Genomics Institute (BGI), Shenzhen, China to develop a *de novo* reference transcriptome from the spathes of ‘Honduras’ cut-flowers. Primary sequence data (paired-end, 91 bp reads) was generated using the Illumina HiSeq 2000 (Illumina, Inc., San Diego, CA). Trinity software [34] with default parameters, was used to assemble reads. The software’s TGICL [35] and Phrap [36] were utilized to get sequences that could not be extended on either end.

Unigenes were aligned with blastx (expect value < 10^−5^) to NCBI non-redundant protein (Nr), Swiss-Prot, Kyoto Encyclopedia of Genes and Genomes (KEGG), and Cluster of Orthologous Groups (COG) databases, to identify proteins with the highest sequence similarity to the respective unigene along with their functional annotation. Proteins with the highest ranks in the BLAST results were used to determine the coding regions in the unigenes and translate them into peptide sequences. To further characterize the function of the genes identified, DEGs and consensus sequences of isoforms were mapped against the UniProtKB/Swiss-Prot database using an expected value threshold of 1x10^-5^. The BlastX output was subjected to Blast2GO for Gene Ontology (GO) analysis.

### Analysis of RNA sequencing data

**Fig 2:**
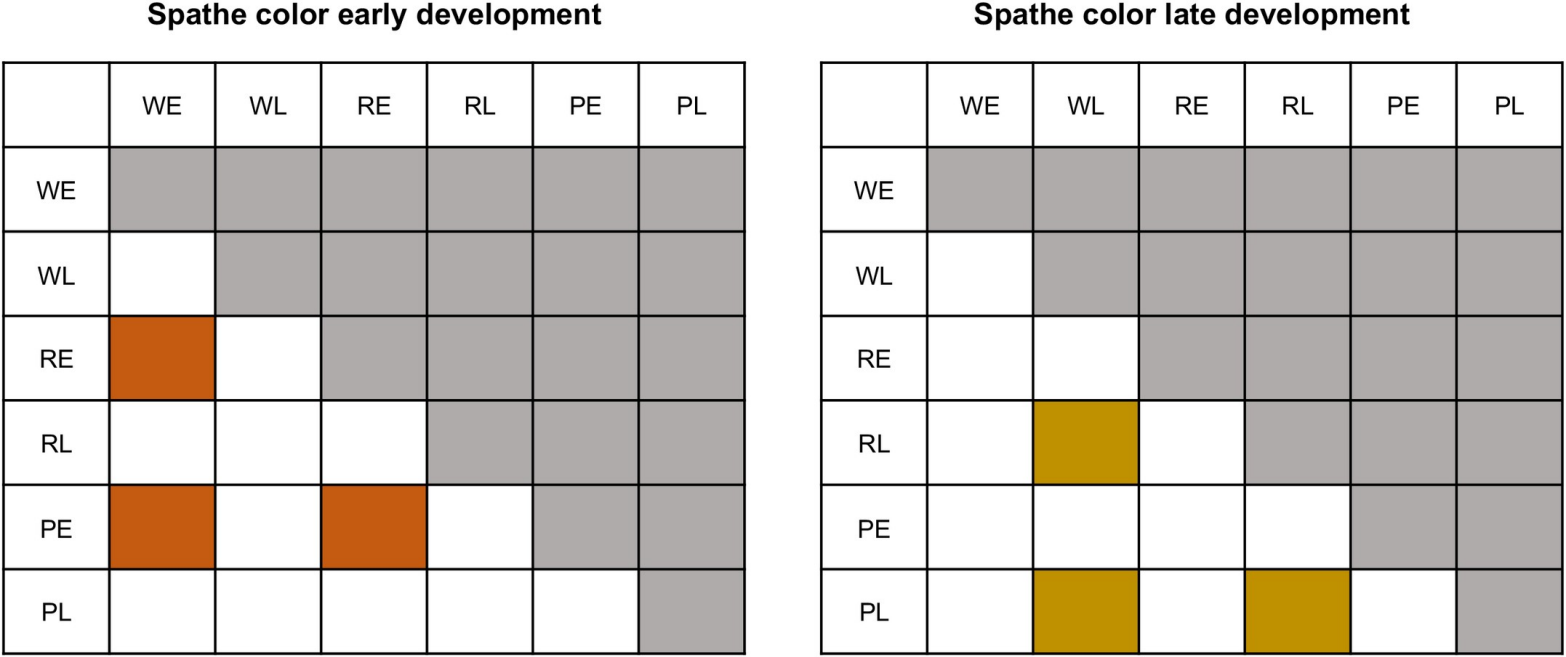
Matrices depicting specific contrasts designed to identify genes differentially expressed. Comparisons between spathes of different colors (W, R, P) in stage 2 (Early (E)); and between spathes of different colors in stage 6 (Late (L)).

Quality per sample was assessed using FastQC v0.11.1 [37]. Trim Galore v0.5.0 and Cutadapt v2.10 [38,39] were used to filter the raw reads. Filters applied included trimming of adapter sequences, removal of reads containing poly-N, and removal of reads with low quality (quality value of over 50% bases of the read was < 5 and error rate of 0.2). Mapping and alignment of reads to transcripts was performed with bowtie2 v2.4.2 [40,41]. All ambiguously mapping positions were maintained to estimate, using a probabilistic approach, the number of reads supporting the expression of any given transcript, as implemented in RSEM [42]. We fitted a generalized linear model with color and stage treatments with a negative binomial using the quasi-likelihood approach to reduce the impact of false positives with the edgeR software [43]. In this model, we tested simultaneously for the effect of developmental stage (E and L) and color (W, R, and P) and controlled for differences in library size. We set two sets of contrasts *a priori*, in which we aimed to identify differential expression between colors at each stage (Fig 2).

Normalized read counts from the top 10,000 variable genes were analyzed with multidimensional scaling (MDS) to visualize the overall variation in the samples using plotMDS in the limma package [44] in R [45]. An initial screening of the data was carried out through a hierarchical clustering dendrogram for each treatment based on the Euclidian distance and grouped with the unweighted pair group method with arithmetic mean (UPGMA) using the dendrogram function of the ape package [46] of R. All code is available at https://github.com/oeco28/Anthurium_differential_expression.

Differentially expressed genes (DEGs) were calculated by using the total number of transcripts per million (TPM) for filtering purposes and using the estimated effective counts of reads mapping to each transcript with the software package edgeR [43,47]. DEGs were defined as genes having a false discovery rate (FDR) [48] < 5x10^-4^ and an absolute log fold change (logFC) ≥ 2. Pearson’s correlations were calculated for all genes with putative connection with the anthocyanin pathway. Gene sets were generated considering those with 0.8 < correlation < -0.8 and *p-value* < 0.001 as significantly co-expressed.

Finally, for the genes that present a function of interest, we took the Log_2_ of the TPM and estimated a centered and normalized measure of expression in the following way:

$$norm(expr)=\left({log}_{2}({TPM}_{i})-\bar{{log}_{2}(TPM)}\right)/se\left({log}_{2}(TPM)\right)$$

where *i* corresponds to individual measure of expression for that gene in individual sample *i*.

All differences in expression for individual genes of interest are shown for these individual genes in the normalized form. All statistical analyses and graphical representations were generated in R.

### Identification of putative transcription factors

An expression network was used to identify potential transcription (specifically MYB-domain TF) responsible for the regulation of differential expression of genes involved in the anthocyanin metabolism. For this, we performed a weighted correlation network analysis (WGCNA) as implemented in WCGNA package [49] in R. Before network construction the proper soft-thresholding power (β) was determined at the inflection point of the curve of the soft threshold (power) vs scale free topology model fit. Followed by a search for modules of potentially co-regulated genes in association with differences in color between spathes. We extracted all of the transcripts found in each module and identified the modules containing genes involved in anthocyanin metabolism that showed significant differential expression (p ≤ 0.0005). Then, we extracted the putative MYB-domain containing genes that were identified as being in the same module that contained DEGs of interest (involved in anthocyanin metabolism) and retained those with the highest correlation in expression.

## Results

### *De novo* transcriptome and sequencing

For the *de novo* transcriptome, a total of 114,334,082 primary sequence reads were obtained from the Illumina sequencer. After stringent quality control checks and filtering, 105,143,382 high-quality clean reads (9,462,904,380 nucleotides) were identified. Among the reads, 98.08% of the nucleotides had quality values > Q20, while the proportion of unknown nucleotides was < 0.00%, and the GC content was 42.97%. Filtered reads were assembled into 151,652 unigenes, the presence of unknown bases in the sequences varying between 0% to 5%, and the length of the unigenes ranged from 201 to 12548 bp.

**Fig 3:**
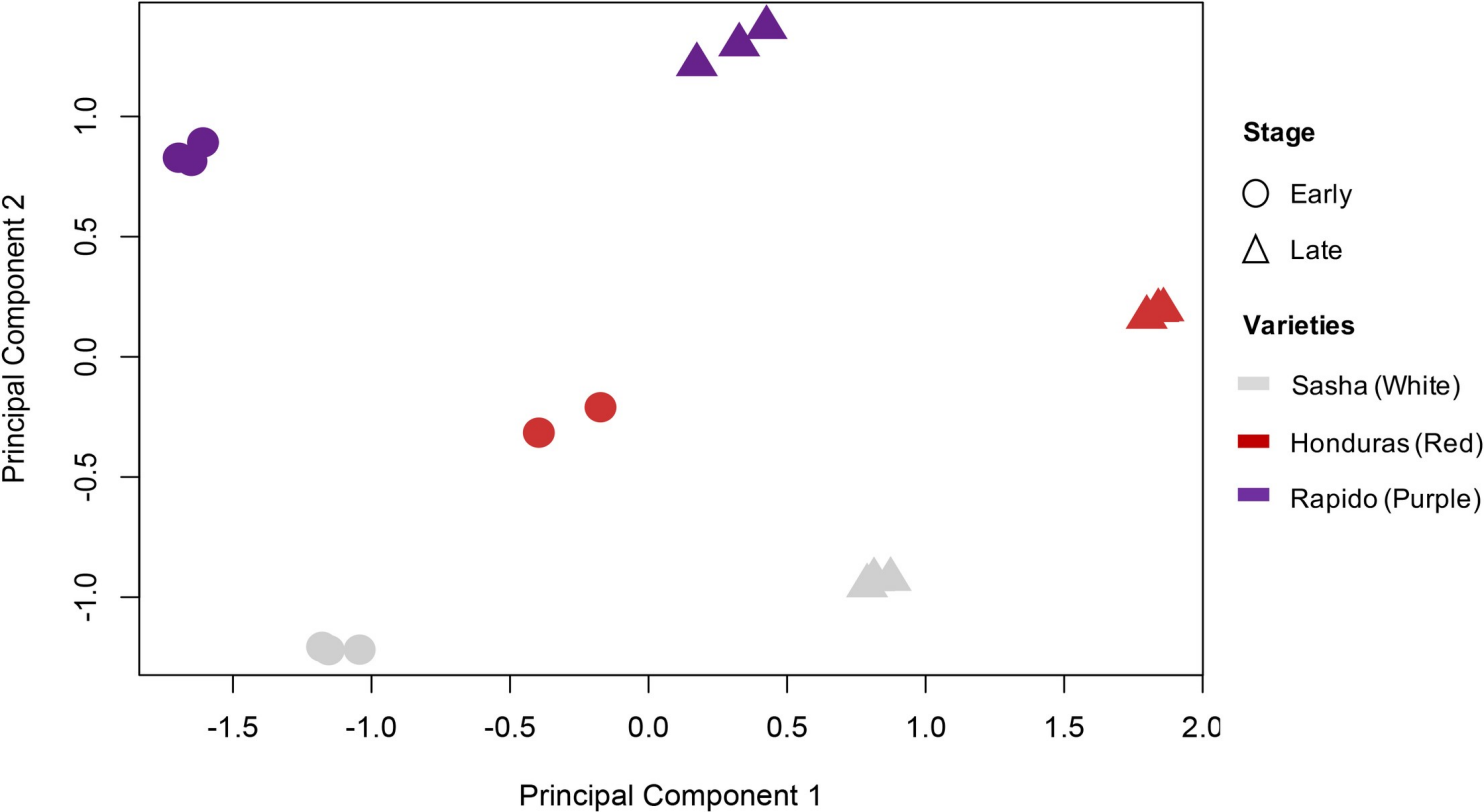
Multidimensional scaling based on gene expression. Samples on the negative scale of principal component 1 correspond to early developmental stages (circles) for either color, and samples on the positive side correspond to late developmental stages (triangles). Dispersion along principal component 2 explained variation between different colors: white (light grey), red (red), and purple (purple).

For the RNA-seq, we obtained a total of 391.29 million clusters (782.58 million reads) of paired-end sequencing with Illumina Phred qualities ≥ Q30. The expected number of reads per sample was 21.78 million reads or 5.5% of the total reads. The distribution of the reads among samples was relatively even across samples, with samples having between 3.8% and 6.7% of the total reads (Additional file 1: S1 Fig). On average, 85% of reads mapped to the reference transcriptome per sample. There was a total of 151,652 transcripts in the reference transcriptome. There were 58,476 transcripts for which the number of mapped reads across samples exceeded a minimum threshold of TPM on average across samples. One of the samples (RE2) was excluded from the analysis because it showed a very low rate of mapping to the reference transcriptome and was a clear outlier based on the UPGMA cluster analysis (Additional file 2: S2 Fig). Finally, the MDS analysis clearly separated the stages in principal component 1 and spathe colors in principal component 2 (Fig 3).

### Differential expression between stages and across different color spathes

**Fig 4:**
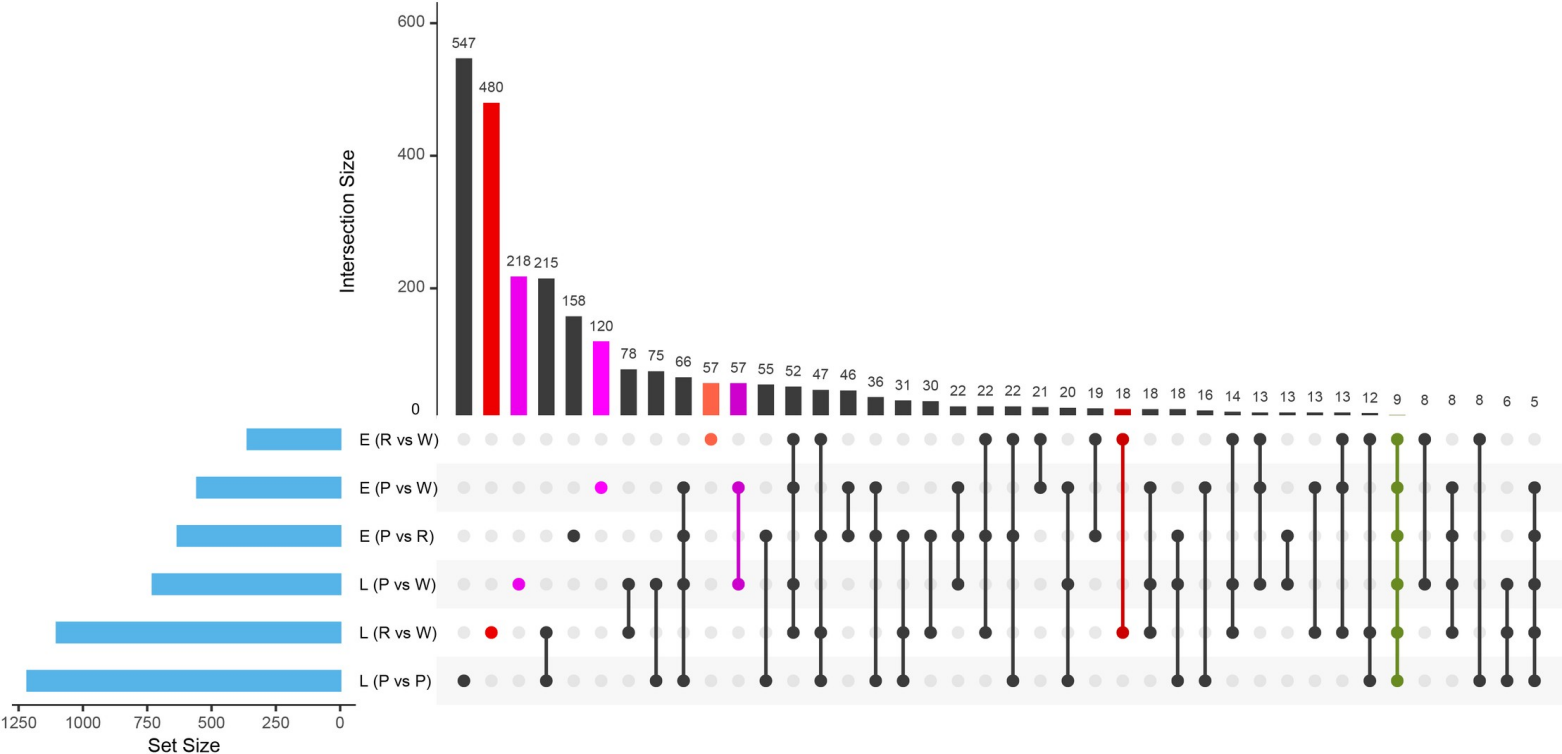
UpSet plot of the differentially expressed genes (DEGs) of RNA-Seq. A quantitative display of the intersection of sets showing the number of differentially expressed genes (FDR threshold value of 5x10^-4^ and logFC ≥ 2) between the comparison of developmental stages and spathe colors.

**Fig 5:**
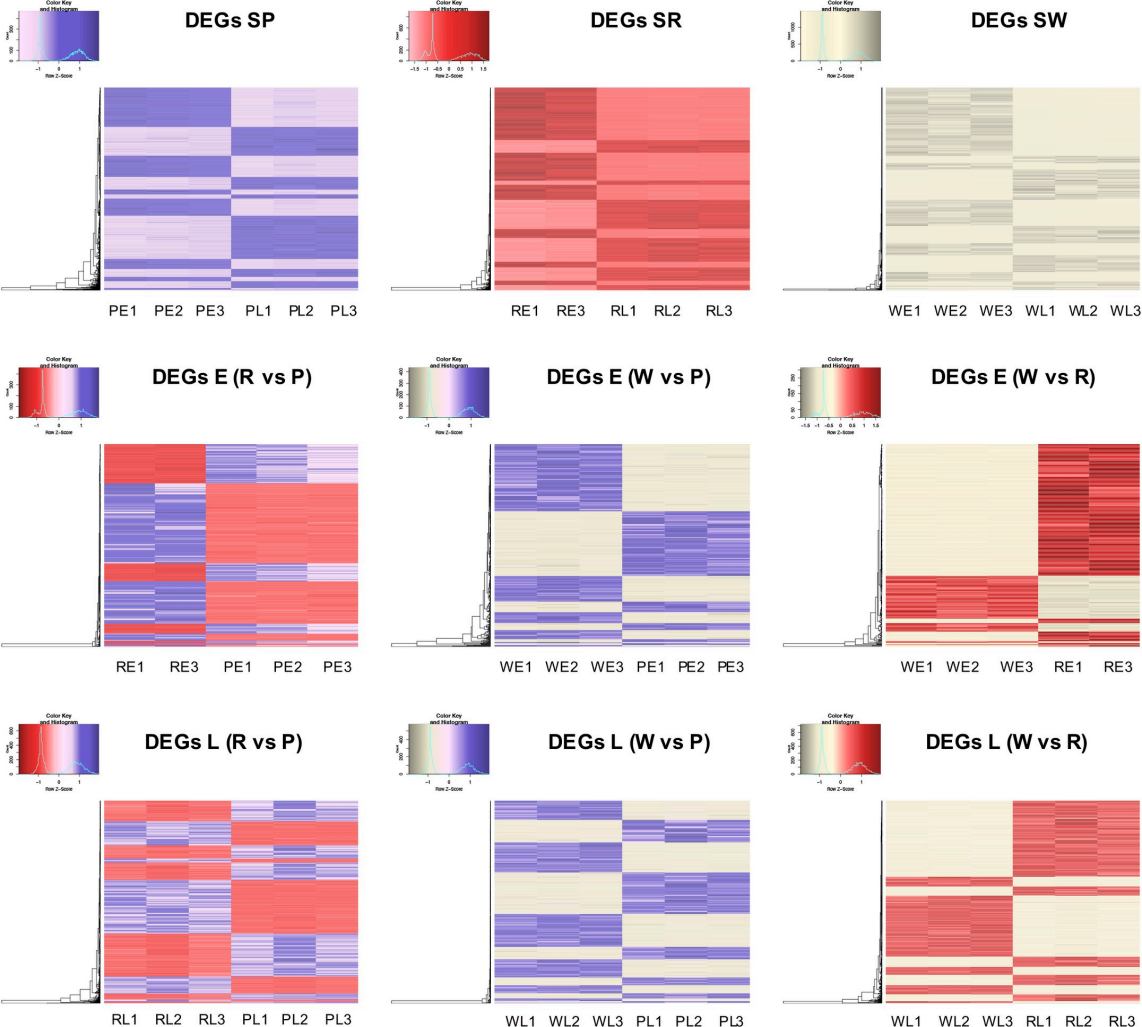
Heat map of RNA-Seq transcriptome analysis for 8,324 selected genes from the *Anthurium andraeanum*. For each illustration: S: stage, W: white, R: red, P: purple; and for stages: E: early, L: late. The biological replicates for each combination of the color stages are indicated with numbers (1,2,3). Only genes with log fold change (logFC) ≥ 2 and at an FDR < 5x10^-4^ are represented in the heatmaps. In figures, lighter colors correspond to under-expressed genes and darker colors to over-expressed genes.

We identified 8,324 DEGs at a conservative FDR threshold value of 5x10^-4^ and logFC ≥ 2 for all the pairwise contrasts after fitting a quasi-likelihood model. There was a similar number of DEGs between stages within each color of the spathe (Table 1 and Fig 4). The relative number of DEGs between colors in the early stage of spathe development was generally smaller than the number of DEGs in the late stage. We found groups of genes showing similar patterns of differential expressions across comparisons (Fig 5).

**Table 1:** Number of genes differentially expressed between stages and spathe colors.

Comparisons †	Genes
EW vs ER	673
EW vs EP	1,088
ER vs EP	1,339
LW vs LR	1,772
LW vs LP	1,308
LR vs LP	2,144

† E: early, L: late, W: white, R: red, P: purple.

Based on annotations, we identified that a large number of genes involved in transcriptional regulation and cell wall expansion were differentially regulated between stages for all colors. The pattern of differential expression was more complex between colors within each developmental stage, which is consistent with the fact that between the cultivars, the spathes were also highly differentiated in shape in addition to color. A comprehensive list of differentially expressed genes for each comparison is described in the additional file 3: S1 Table. Quantitative set diagrams showed how DEGs were shared across comparisons for each set of contrasts (Fig 5). In our stage comparison (E vs L), we observed for all colors a general trend of having more statistically significant downregulated genes than upregulated in the late stage. Although we did not find a remarkable difference between the number of downregulated and upregulated genes in the rest of the comparisons, the general trend suggested that there was a slightly larger number of downregulated genes in colored spathes (R, P) when compared to the white (W) spathe at both development stages (E, L) (Additional file 3: S1 Table).

**Fig 6:**
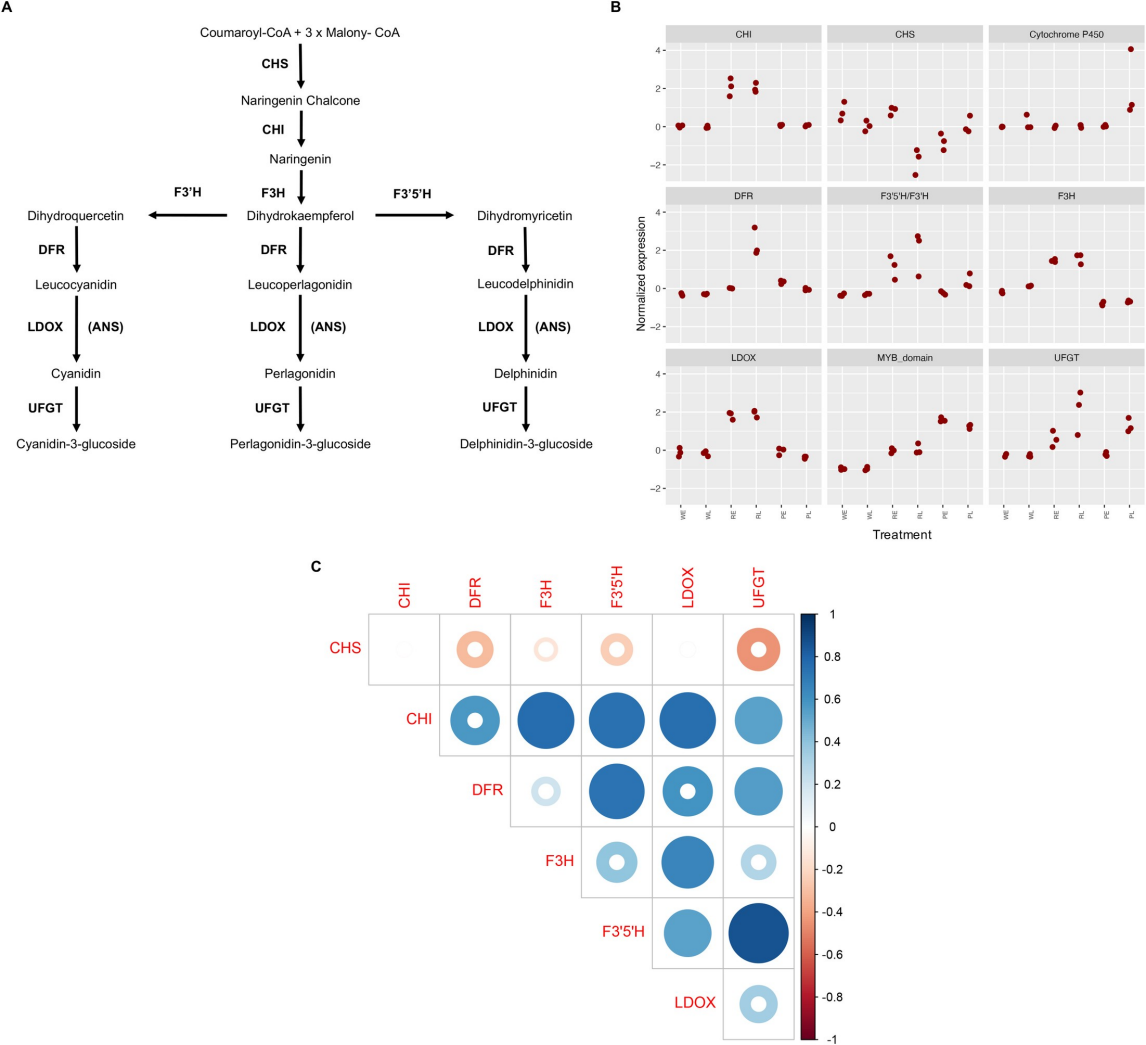
Differential expression of genes in anthocyanin pathway. (A) Adapted diagram showing the anthocyanin biosynthetic pathway. *CHS*: chalcone synthase; *CHI*: chalcone isomerase; *F3H*: flavanone 3-hydroxylase; *F3’H*: flavonoid 3’-hydroxylase; *F3’5’H*: flavonoid-3’,5’-hydroxylase; *DFR*: dihydroflavonol 4-reductase; *LODX-ANS*: anthocyanidin synthase; *UFGT*: UDP-Glc-flavonoid 3-O-glucosyl transferase; and *FLS*: flavonol synthase. Modified from Holton and Cornish (1995). (B) Measure of expression based on Log2 of transcripts per million (TPM). (C) Pearson correlation analysis between the genes related with the anthocyanin pathway. Complete colored circle indicates highly significance.

We found a large number of DEGs in our comparisons, but we focused our attention to those genes involved in the anthocyanin biosynthetic pathway (Fig 6A, Table 2). We found that the *CHI* gene is highly expressed in the red cultivar for both stages in comparison with the other samples. The *CHS* gene, at the top of the regulatory cascade of the anthocyanin biosynthetic pathway, had higher expression levels in the white spathe in both stages of development and for the red early stage; however, for this gene, the red late, the early and late stages of purple cultivar presented lower expression. The gene related with the cytochrome *P450* presented higher expression in the late stage of purple. The *DFR* gene presented higher expression for the red cultivar on stage late compared to the other stages in the other two cultivars. The genes *F3’5’H*, *F3H*, and *LDOX* presented the higher expression in both stages of the red cultivar (Fig 6B, Table 2) compared to the white and purples cultivars. In addition, a putative MYB-domain was highly expressed in the purple stages, following by the red ones and finally down expressed in the white cultivar. Details on the identification of this putative MYB-domain are provided below in section Identification of putative transcription factors. Finally, the *UFGT* gene presented higher expression in the purple and red cultivars compared to the white one.

**Table 2:** Differential expression of genes in the anthocyanin pathway between White and Red (WR), White and Purple (WP), and Red and Purple (RP) at early (E) or late (L) stages.

Gene	Comparison	LogFC	FDR
***CHI***	**E_WR**	10.54	1.5E-03^&^
	**L_WR**	10.83	4.23E-05*
	**E_WP**	0	1.00
	**L_WP**	0	1.00
	**E_RP**	-10.54	9.87E-05*
	**L_RP**	-10.83	3.98E-05*
***CHS***	**E_WR**	-0.11	0.91
	**L_WR**	-3.13	1.48E-05*
	**E_WP**	-0.45	0.3804103
	**L_WP**	-0.52	0.28
	**E_RP**	-0.34	0.54
	**L_RP**	2.62	5.24E-05*
***F3H***	**E_WR**	1.48	5.18E-06*
	**L_WR**	1.08	1.03E-05*
	**E_WP**	-1.49	2.17E-06*
	**L_WP**	-1.57	8.86E-07*
	**E_RP**	-2.97	1.58E-08*
	**L_RP**	-2.65	1.28E-08*
***F3’H/F3’H***	**E_WR**	7.2	2.88E-05*
	**L_WR**	8.93	2.72E-06*
	**E_WP**	3.68	6.10E-03^&^
	**L_WP**	7.3	2.42E-05*
	**E_RP**	-3.52	4.80E-04*
	**L_RP**	-1.63	1.35E-02
***DFR***	**E_WR**	4.78	8.05E-05*
	**L_WR**	8.29	2.30E-07*
	**E_WP**	5.7	5.02E-06*
	**L_WP**	4.93	3.68E-05*
	**E_RP**	-3.28	7.89E-04^&^
	**L_RP**	-3.36	1.29E-05*
***UFGT***	**E_WR**	5.51	8.42E-04*
	**L_WR**	7.38	2.78E-05*
	**E_WP**	1.32	5.04E-01*
	**L_WP**	6.82	8.25E-05*
	**E_RP**	-4.19	1.54E-03^&^
	**L_RP**	-0.57	4.62E-01
***LDOX***	**E_WR**	1.48	5.18E-06*
	**L_WR**	1.08	1.03E-05*
	**E_WP**	-1.49	2.17E-06*
	**L_WP**	-1.57	8.86E-07*
	**E_RP**	-2.97	1.58E-08*
	**L_RP**	-2.65	1.28E-08*

* Significant differences at the a priori set threshold of 5.00E-04.

^&^ Differential expression values with FDR between 1.00E-02 and 4.99E-04.

The Log Fold change (LogFC) and False Discovery Rate (FDR) are presented.

We analyzed the co-expression between genes in the anthocyanin pathway by estimating the correlation of normalized TPMs across the different samples. Using a minimal TPM value of 5 as a threshold, the Pearson coefficient heatmap (Fig 6C) suggests at least 6 highly correlated groups. A clear pattern emerges from this analysis showing that the *CHI* gene is highly positively correlated with other downstream genes in the anthocyanin pathway. Other genes showing an interesting correlation pattern of expression are *F3’5’H/F3’H*, *F3H*, and *DFR*.

### Identification of putative transcription factors

**Fig 7:**
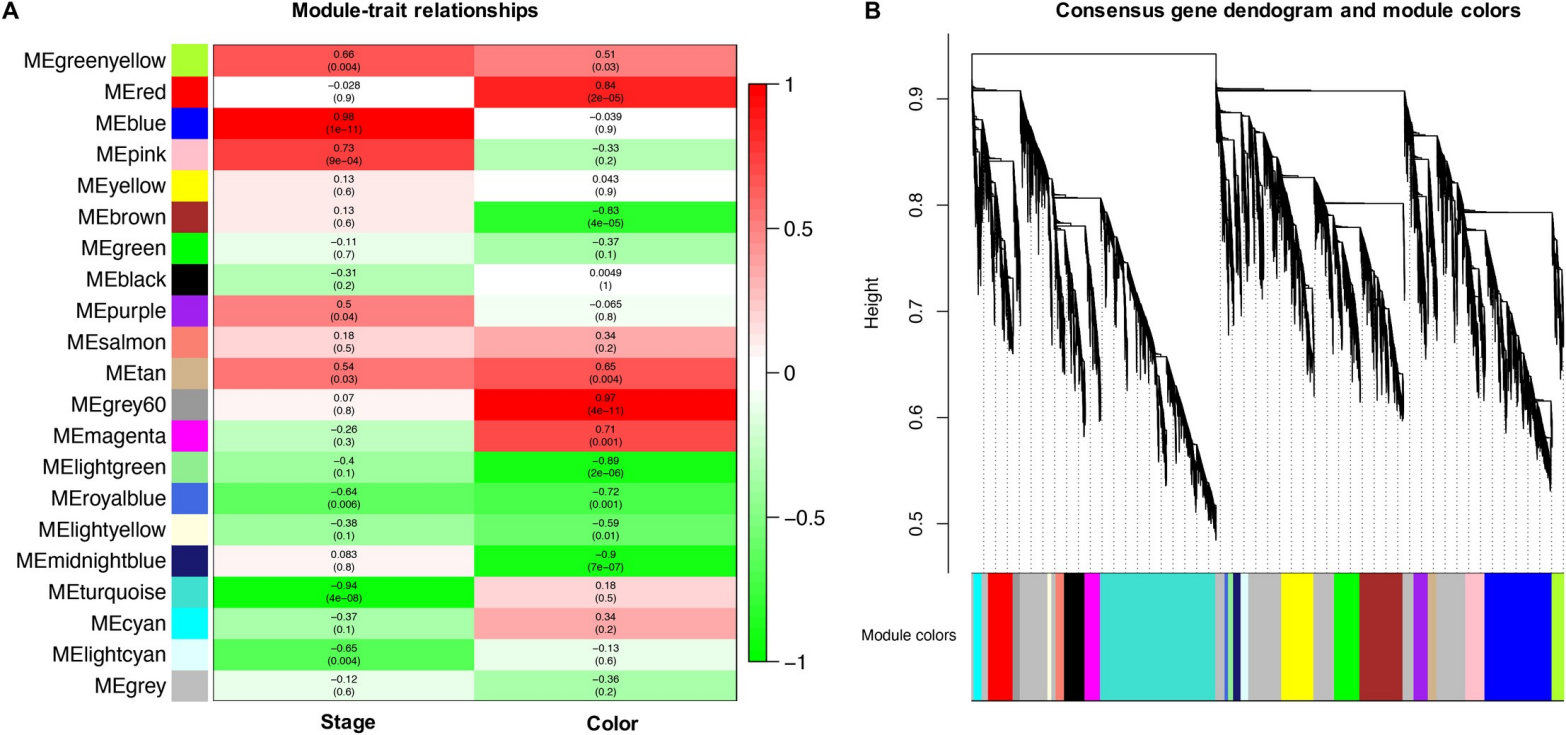
Weighted correlation network analysis of expression across all samples. (A) module-trait correlations and corresponding *p*-values (in parentheses). Each row corresponds to a module eigengene (ME), and each column to a trait. The color scale on the right shows module-trait correlations from − 1 (green) to + 1 (red). (B) Hierarchical cluster tree showing 21 modules of co-expressed genes. Each of the 8,324 DEGs is represented by a leaf in the tree, and each of the modules by a major tree branch. The lower panel shows modules in designated colors.

It has been shown that tissue-specific regulation of *CHS/CHI* and other relevant genes in the anthocyanin pathway is controlled by a MYB-like domain protein in different systems [18–20]. The *Anthurium* transcriptome, and many plant genomes have a large number of annotated MYB-domain and MYB-like domain proteins (Additional file 4: S2 Table). In order to reduce the number of potential candidates that could be involved in the control of *CHI* differential expression, we performed an expression network analysis across all samples using WGCNA. The soft-thresholding power and connectivity between DEGs, were screened prior to the WGCNA module analysis. The soft threshold power of 6 (β  =  6) was selected according to the preconditions of approximate scale-free topology. The general results of the modules and clusters of genes showing similar patterns of expression are shown in Fig 7A and 7B. Our analysis suggested that 21 modules or clusters of genes with similar expression can explain the diversity of gene expression profiles across spathe developmental stages and colors. A large number of transcripts were assigned to a module with low assignment scores (grey, n Genes = 10,998) indicative of genes that cannot follow a characteristic expression profile. Gene clusters varied in size from 557 (light cyan) to 8774 (turquoise). The large number of genes present in a moderate number of clusters, for such a divergent set of plants, suggested that changes in color across developmental stage was a tightly controlled process.

We focused our attention on the red cluster containing the *CHI* gene (Unigene109494) and used it to identify MYB-like domain proteins that showed similar patterns of expression (Additional file 5: S3 Table). Our analyses showed that this cluster contained nine MYB-domain proteins encoding genes, that showed a highly significantly correlated transcriptional profile (modular correlation r2 > 0.8 and p < 0.0001)_compared to *CHI* (Fig 7A). Out of these, three MYB encoding genes putatively produced proteins larger than 300 aa, and one of them (Unigene7734) has a target regulatory sequence for miRNA recognition.

## Discussion

The large diversity in anthocyanin pigmentation in flowers has been an important feature in the coevolution of plants and pollinators. Specifically, for *A*. *andraeanum*, it has been already established that the spathes accumulate anthocyanins progressively, reaching large quantities at stage 3 and above, and producing other flavonoids [27,50]. More recent work performed in dark red, red, light red, white, orange, and coral cultivars of *Anthurium*, suggested that *F3’H* expression might be a key control point in the regulation of anthocyanin biosynthesis and is strongly associated to the intensity of pink color [51,52]. In concordance, Collette et al., (2004) [29] established that high levels of *CHS* and *F3H* genes during the early stages of development could generate the differences between colors. Although previous analyses have implicated *CHS* among other enzymes in the biosynthetic pathway in the determination of color in spathe in *Anthurium*, none have been conclusive. For this reason, to gain insight into the molecular events that regulate the spathe color in *A*. *andraeanum*, we used RNA-seq and WGCNA analysis to examined the expression patterns of various structural genes (*CHI*, *CHS*, *F3H*, *F3’5’H/F3’H*, *DFR*, and *ANS*) to determine differences in color in plants with white, red and purple spathes over different developmental stages. We divided the variable genes into co-expression modules by unsigned network construction based on their expression patterns to identify putative TFs like MYB that could help the understanding of the color in this species.

In this work, we generated a new *de novo* reference transcriptome for *A*. *andreanum* L. from a standard and established variety, Honduras, well known among growers and producers. We anticipate that this will be a resource that will be broadly used by growers interested in characterizing transcriptional profiles for the species. We identified differentially expressed genes among three cultivars to identify genes involved in the determination of color on spathes in *A*. *andreanum* L. The *CHS* gene was the main regulator differentially down-regulated in the red late and early and late stages of purple spathe when compared to the white spathe. The *CHS* gene followed a profile of expression in red spathes characterized by higher levels of expression in early stages followed by a subsequent decay. This result was surprising, and we hypothesize that the decline in expression of *CHS* in red spathe could be due to the fact that this gene catalyzes the first reaction in the anthocyanin biosynthesis, but is not needed in later stages to produce Naringenin Chalcone in higher proportion compared to early stages. Similar results consistent with the behavior of *CHS* content across stages have been reported previously in other species [53,54]. However, CHS seems to present different profiles of expression in time, suggesting a different role in different systems, like petunia flowers where the expression of *CHS* increases as development progresses [55]. Our results suggest that *CHS* plays a role in the biosynthesis of flavonoid in *A*. *andraeanum* and in the differentiation between white and colored spathes. In contrast with previous studies, we identified the importance of the *CHI* gene due to its highly correlated expression. In other species like tobacco [56] and tomato [57], it has been demonstrated that manipulating the *CHI* caused changes in the anthocyanin content as well as flower, peels, and flesh color intensity. Additionally, findings from other studies show that genes implicated with *CHI* are coordinately expressed with *F3’H* in the anthocyanin biosynthetic pathway [58]. Studies also show that the coordinated expression for these genes becomes more evident as transcript levels increase with plant maturity [58]. Taken together, the profiles of expression of CHI and F3’H, suggests that their coordinated expression plays an important role in the determination of color in *A*. *andraeanum*, *e*specially when contrasting red and white spathe varieties. The high positive correlation found between *CHI* and other genes in the pathway (Fig 6C) is consistent with previous findings in other systems [59–61]. Our analysis suggests that differences in color are likely to be driven by differential expression of multiple in the anthocyanin pathways that work coordinately to favor the production of pigments.

We revealed that most of the structural genes (*CHI*, *F3H*, *F3´5´H/F3’H*, *DFR*, and *UFGT*) are involved in the anthocyanin biosynthetic pathway were significantly down-regulated in the white flowers. We detected significant differential expression of the *DFR* gene between white and red. Our analyses strongly suggests that *DFR* plays a key role in the formation of anthocyanins, which have been reported to directly determines the development of pink or white color in tobacco flowers [62]. This result suggested, that the expression of *DFR* could contribute to the color differences between the white and colored spathes in this study one the developmental stage is advanced. Similar findings have also been noted by Gopaulchan et al. (2014) [51], where the white spathes displayed equivalent levels of *DFR* transcript to the red and orange spathes at stage 2, and had higher expression at stage 6 compared to the colored spathes. In contrast, the *UFGT* genes were strictly inhibited in white flowers. It has been reported that the down-regulation of McDFR in apple fruit reduced the expression levels of some structural genes (*F3H*, *F3’H*, *DFR*, *ANS*, and *UFGT*), while the *CHS* and *CHI* genes were up-regulated [63]. It indicates that the altered expression of DFR also affected the expression of other anthocyanin biosynthetic genes. In this study, interestingly, reveals that *CHI* genes are the most significantly up-regulated genes in the comparison between white and red, which seem to be one of the important candidate genes in determining the flower color. Because of the great similarity between *F3’5’H* and *F3’H*, we couldn’t distinguish between those two genes in the *de novo* transcriptome using amino acid sequence alone. Yet, the expression results showing a significant over-expression of this gene in red cultivar when compared to white, but not in the purple to white, strongly suggests that this gene is the *F3’H* gene driving the pathway towards the production of red pigment.

We find compelling evidence that the determination of purple color at late stages might be regulated by increased expression of *F3’5’H/F3’H*, cytochrome *P450*, MYB-domain, and *UFGT*. More importantly, in the purple spathe, we identified a cytochrome *P450* oxidase differentially up-regulated when compared to red colored spathe which appeared to determine the difference between the red and purple hues. Functional annotation analysis suggested that the cytochrome *P450* gene may encode an additional *F3’H* or *F3’5’H genes*. This latter result was striking because initially the purple spathe displays a red hue at the early stage of spathe development and then acquires the purple hue as it develops into a mature spathe progresses and this cytochrome *P450* gene could be a highly divergent *F3’H* or *F3’5’H* gene. Hence, the *CHS* gene may have a stronger influence on anthocyanin accumulation than other structural genes, at least in the early stages of ‘Honduras’ and ‘Rapido’ cultivars. Taken together, our results suggest that *F3’H* is more likely to be a key structural gene in the late stages of the purple spathe. Besides, the accumulation of higher amounts of anthocyanin in purple (Rapido cultivar) may also be due to a cumulative effect of the high expression levels of all the other structural genes like *P450*. Previous studies in other species indicated that a single gene is not responsible for anthocyanin accumulation and that anthocyanin biosynthesis involves the coordinated mechanism of many genes [64,65]. However, additional research is necessary to test this hypothesis in *A*. *andraeanum*.

In this paper, a weighted gene co-expression network (WGCNA) analysis was also performed in order to identify other genes involved in anthocyanin accumulation in *Anthurium*. WGCNA is a method for analyzing the gene expression patterns of multiple samples. Genes with similar expression patterns can be clustered, moreover, the relationship between modules, allowing to obtain information on both genes function and the mechanisms controlling the traits of interest [66]. This method has been recently used to dissect anthocyanin pathway in apricots [67], apples [68] and eggplant [69].

Among the genes present in each weighted module that correlated with the metabolite of interest, we found 9 DEGs coding for transcription factors, indicating that there is a strong relationship between the *CHI* gene and MYB-domain, as previously hypothesized, even if the mechanism of this connection is still unclear.

Analyses in different plant systems have focused on understanding the pattern of expression of specific genes and demonstrated how diverse these underlying changes can manifest in differences in coloration can be [70–72]. The importance of R2R3 MYB TF in the co-regulation of the anthocyanin biosynthetic pathway have been demonstrated in other species. For example, in *Paeonia ostia*, a study found that two TF, PoMYB2, and PoSPL1, seem to negatively regulate anthocyanin accumulation by interfering with the formation of the MYB-bHLH-WDR complex [73]. Besides, analyses in *Petunia hybrida* have shown that two bHLH TFs interacts with a MYB and WD repeat protein to regulate the expression of *CHS* and *DFR* genes [17,50,74–76]. In our study, the use of WGCNA to identify co-expression patterns among the six different tissues was especially key to identifying potential regulatory elements for the pathways of interest. Additionally, the mRNA encoding the putative MYB-domain contained a silencing RNA-target motif that was polymorphic among samples of white and colored spathes. At this point, the identification of this MYB-domain putative regulator remains hypothetical and further studies are needed to confirm its implication in the regulation of color in *A*. *andraeanum*.

## Conclusions

*A*. *andraeanum* is a monocot plant type that presents a spathe with highly diverse pigmented colors. The present study showed that differences between the three colored spathes as well as differences among developmental stages could be explained by differences in their relative gene’s expression abundances. Furthermore, could be explained due to coordinated expression between the genes *CHI*, *DFR*, *F3H*, and *F3’5’H*. There is also evidence from our study that the accumulation of higher amounts of anthocyanin in the purple spathe could be due to additional regulatory cumulative effect of the high expression levels of structural genes like the cytochrome *P450*. While the correlation between gene expression provides preliminary evidence of the role of these genes in determining spathe color intensity, further work is required to elucidate the roles of these genes.

## Supporting information

S1 Fig Distribution of reads among samples. (TIF)

S2 Fig Hierarchical clustering analysis of DEGs. (TIF)

S1 Table Differential expressed genes between developmental stages and spathe color in *Anthurium andraeanum*. (XLSX)

S2 Table Functional annotation of transcripts of *Anthurium andraeanum*. (TXT)

S3 Table Cluster of genes according to module colors performed with the weighted correlation network analysis of expression across all samples. (XLSX)

## References

[1] Sobral M, Veiga T, Domínguez P, Guitián JA, Guitián P, Guitián JM. Selective pressures explain differences in flower color among *Gentiana lutea* populations. PLoS One. 2015;10: 1–15. doi: 10.1371/journal.pone.0132522 26172378 PMC4501686

[2] Hirota SK, Nitta K, Suyama Y, Kawakubo N, Yasumoto AA, Yahara T. Pollinator-mediated selection on flower color, flower scent and flower morphology of Hemerocallis: Evidence from genotyping individual pollen grains on the stigma. PLoS One. 2013;8: 1–11. doi: 10.1371/journal.pone.0085601 24376890 PMC3871637

[3] Hopkins R, Rausher MD. Pollinator-mediated selection on flower color allele drives reinforcement. Science (80-). 2012;335: 1090–1092. doi: 10.1126/science.1215198 22300852

[4] Jones KN, Reithel JS. Pollinator-mediated selection on a flower color polymorphism in experimental populations of *Antirrhinum* (scrophulariaceae). Am J Bot. 2001;88: 447–454. doi: 10.2307/2657109 11250822

[5] Kamemoto H, Kuehnle AR. Breeding anthuriums in Hawaii. University of Hawaii Press; 1996. Available: http://books.google.com/books?id=xhLDJEup0JMC&pgis=1.

[6] Kamemoto H, Iwata RY, Marutani M. Genetics of the major spathe colors in anthuriums. Research series—Hawaii Agricultural Experiment Station, Hitahr College of Tropical Agriculture and Human Resources (USA). 1988.

[7] Wannakrairoj S, Kamemoto H. Inheritance of purple spathe in *Anthurium*. J Am Soc Hortic Sci jashs. 1990;115: 169–171.

[8] Dufour L, Guérin V. Growth, developmental features and flower production of *Anthurium andreanum* Lind. in tropical conditions. Sci Hortic (Amsterdam). 2003;98: 25–35. doi: 10.1016/S0304-4238(02)00196-6

[9] Khoo HE, Azlan A, Tang ST, Lim SM. Anthocyanidins and anthocyanins: colored pigments as food, pharmaceutical ingredients, and the potential health benefits. Food Nutr Res. 2017;61: 1361779. doi: 10.1080/16546628.2017.1361779 28970777 PMC5613902

[10] Santos-Buelga C, Mateus N, Freitas V. Anthocyanins. Plant Pigments and Beyond. J Agric Food Chem. 2014;62. doi: 10.1021/jf501950s 24970106

[11] Tanaka Y, Sasaki N, Ohmiya A. Biosynthesis of plant pigments: Anthocyanins, betalains and carotenoids. Plant J. 2008;54: 733–749. doi: 10.1111/j.1365-313X.2008.03447.x 18476875

[12] Li D, Wang P, Luo Y, Zhao M, Chen F. Health benefits of anthocyanins and molecular mechanisms: Update from recent decade. Crit Rev Food Sci Nutr. 2017;57: 1729–1741. doi: 10.1080/10408398.2015.1030064 26192537

[13] Pojer E, Mattivi F, Johnson D, Stockley CS. The Case for Anthocyanin Consumption to Promote Human Health: A Review. Compr Rev Food Sci Food Saf. 2013;12: 483–508. doi: 10.1111/1541-4337.12024 33412667

[14] Elibox W, Umaharan P. Inheritance of major spathe colors in *Anthurium andraeanum* Hort. is determined by three major genes. HortScience. 2008;43: 787–791. doi: 10.21273/hortsci.43.3.787

[15] Holton TA, Cornish EC. Genetics and biochemistry of anthocyanin biosynthesis. Plant Cell. 1995;7: 1071–1083. doi: 10.1105/tpc.7.7.1071 12242398 PMC160913

[16] Springob K, Nakajima J, Yamazaki M, Saito K. Recent advances in the biosynthesis and accumulation of anthocyanins. Nat Prod Rep. 2003;20: 288–303. doi: 10.1039/b109542k 12828368

[17] Quattrocchio F, Baudry A, Lepiniec L, Grotewold E. The regulation of flavonoid biosynthesis BT—the science of flavonoids. In: Grotewold E, editor. New York, NY: Springer New York; 2006. pp. 97–122. doi: 10.1007/978-0-387-28822-2_4

[18] Lin-wang K, Bolitho K, Grafton K, Kortstee A, Karunairetnam S, Mcghie TK, et al. An R2R3 MYB transcription factor associated with regulation of the anthocyanin biosynthetic pathway in Rosaceae. BMC Plant Biol. 2010;10: 1–17. doi: 10.1186/1471-2229-10-1 20302676 PMC2923524

[19] Motamayor JC, Mockaitis K, Schmutz J, Haiminen N, Livingstone D, Cornejo O, et al. The genome sequence of the most widely cultivated cacao type and its use to identify candidate genes regulating pod color. Genome Biol. 2013;14: r53. doi: 10.1186/gb-2013-14-6-r53 23731509 PMC4053823

[20] Singh R, Low ETL, Ooi LCL, Ong-Abdullah M, Nookiah R, Ting NC, et al. The oil palm VIRESCENS gene controls fruit colour and encodes a R2R3-MYB. Nat Commun. 2014;5: 1–8. doi: 10.1038/ncomms5106 24978855 PMC4078410

[21] Li C, Qiu J, Yang G, Huang S, Yin J. Isolation and characterization of a R2R3-MYB transcription factor gene related to anthocyanin biosynthesis in the spathes of *Anthurium andraeanum* (Hort.). Plant Cell Rep. 2016;35: 2151–2165. doi: 10.1007/s00299-016-2025-8 27424029

[22] Cao H, Wang J, Dong X, Han Y, Ma Q, Ding Y, et al. Carotenoid accumulation affects redox status, starch metabolism, and flavonoid/anthocyanin accumulation in citrus. BMC Plant Biol. 2015;15: 1–16. doi: 10.1186/s12870-014-0410-4 25644332 PMC4323224

[23] Lou Q, Liu Y, Qi Y, Jiao S, Tian F, Jiang L, et al. Transcriptome sequencing and metabolite analysis reveals the role of delphinidin metabolism in flower colour in grape hyacinth. J Exp Bot. 2014;65: 3157–3164. doi: 10.1093/jxb/eru168 24790110 PMC4071837

[24] Mori M, Kondo T, Yoshida K. Anthocyanin components and mechanism for color development in blue veronica flowers. Biosci Biotechnol Biochem. 2009;73: 2329–2331. doi: 10.1271/bbb.90349 19809174

[25] Tan J, Wang M, Tu L, Nie Y, Lin Y, Zhang X. The flavonoid pathway regulates the petal colors of cotton flower. PLoS One. 2013;8: e72364. doi: 10.1371/journal.pone.0072364 23951318 PMC3741151

[26] Zhao D, Tao J. Recent advances on the development and regulation of flower color in ornamental plants. Front Plant Sci. 2015;6: 1–13. doi: 10.3389/fpls.2015.00001 25964787 PMC4410614

[27] Williams CA, Harborne JB, Mayo SJ. Anthocyanin pigments and leaf flavonoids in the family araceae. Phytochemistry. 1981;20: 217–234. 10.1016/0031-9422(81)85096-0.

[28] Li C, Qiu J, Huang S, Yin J, Yang G. AaMYB3 interacts with AabHLH1 to regulate proanthocyanidin accumulation in *Anthurium andraeanum* (Hort.)—another strategy to modulate pigmentation. Hortic Res. 2019;6: 1–16. doi: 10.1038/s41438-018-0066-6 30603098 PMC6312548

[29] Collette VE, Jameson PE, Schwinn KE, Umaharan P, Davies KM. Temporal and spatial expression of flavonoid biosynthetic genes in flowers of *Anthurium andraeanum*. Physiol Plant. 2004;122: 297–304. doi: 10.1111/j.1399-3054.2004.00402.x

[30] Yang Y, Chen X, Xu B, Li Y, Ma Y, Wang G. Phenotype and transcriptome analysis reveals chloroplast development and pigment biosynthesis together influenced the leaf color formation in mutants of *Anthurium andraeanum* ‘Sonate.’ Front Plant Sci. 2015;6: 1–16. doi: 10.3389/fpls.2015.00001 25814997 PMC4356079

[31] Zhang H, Tian H, Chen M, Xiong J, Cai H, Liu Y. Transcriptome analysis reveals potential genes involved in flower pigmentation in a red-flowered mutant of white clover (*Trifolium repens* L.). Genomics. 2018;110: 191–200. doi: 10.1016/j.ygeno.2017.09.011 28966045

[32] Garzón-Martínez GA, Zhu ZI, Landsman D, Barrero LS, Mariño-Ramírez L, Garzon G, et al. The *Physalis peruviana* leaf transcriptome: assembly, annotation and gene model prediction. BMC Genomics. 2012;13: 151. doi: 10.1186/1471-2164-13-151 22533342 PMC3488962

[33] Thomas J, Kim HR, Rahmatallah Y, Wiggins G, Yang Q, Singh R, et al. RNA-seq reveals differentially expressed genes in rice (*Oryza sativa*) roots during interactions with plant-growth promoting bacteria, Azospirillum brasilense. PLoS One. 2019;14: e0217309. Available: doi: 10.1371/journal.pone.0217309 31120967 PMC6532919

[34] Grabherr MG, Haas BJ, Yassour M, Levin JZ, Thompson DA, Amit I, et al. Full-length transcriptome assembly from RNA-Seq data without a reference genome. Nat Biotechnol. 2011;29: 644–652. doi: 10.1038/nbt.1883 21572440 PMC3571712

[35] Pertea G, Huang X, Liang F, Antonescu V, Sultana R, Karamycheva S, et al. TIGR Gene Indices clustering tools (TGICL): a software system for fast clustering of large EST datasets. Bioinformatics. 2003;19: 651–652. doi: 10.1093/bioinformatics/btg034 12651724

[36] Vogel JP, Gu YQ, Twigg P, Lazo GR, Laudencia-Chingcuanco D, Hayden DM, et al. EST sequencing and phylogenetic analysis of the model grass Brachypodium distachyon. Theor Appl Genet. 2006;113: 186–195. doi: 10.1007/s00122-006-0285-3 16791686

[37] Andrews S, Krueger F, Segonds-Pichon A, Biggins L, Krueger C, Wingett S. FastQC: a quality control tool for high throughput sequence data. Babraham, UK: Babraham Institute; 2012 [cited 25 Aug 2019]. Available: http://www.bioinformatics.bbsrc.ac.uk/projects/fastqc/.

[38] Krueger F. Trim Galore. 2018 [cited 9 Jul 2019]. Available: http://www.bioinformatics.babraham.ac.uk/projects/trim_galore/.

[39] Martin M. CUTADAPT removes adapter sequences from high-throughput sequencing reads. EMBnet.journal. 2011;17. doi: 10.14806/ej.17.1.200

[40] Langmead B, Trapnell C, Pop M, Salzberg SL. Ultrafast and memory-efficient alignment of short DNA sequences to the human genome. Genome Biol. 2009;10: R25. doi: 10.1186/gb-2009-10-3-r25 19261174 PMC2690996

[41] Langmead B, Salzberg SL. Fast gapped-read alignment with Bowtie 2. Nat Methods. 2012;9: 357–359. doi: 10.1038/nmeth.1923 22388286 PMC3322381

[42] Haas BJ, Papanicolaou A, Yassour M, Grabherr M, Blood PD, Bowden J, et al. De novo transcript sequence reconstruction from RNA-seq using the Trinity platform for reference generation and analysis. Nat Protoc. 2013;8: 1494–1512. doi: 10.1038/nprot.2013.084 23845962 PMC3875132

[43] McCarthy DJ, Chen Y, Smyth GK. Differential expression analysis of multifactor RNA-Seq experiments with respect to biological variation. Nucleic Acids Res. 2012/01/28. 2012;40: 4288–4297. doi: 10.1093/nar/gks042 22287627 PMC3378882

[44] Ritchie ME, Phipson B, Wu D, Hu Y, Law CW, Shi W, et al. limma powers differential expression analyses for RNA-sequencing and microarray studies. Nucleic Acids Res. 2015;43: e47–e47. doi: 10.1093/nar/gkv007 25605792 PMC4402510

[45] R Core Team, R development core team. R: a language and environment for statistical computing. Vienna, Austria: R Foundation for Statistical Computing; 2008. Available: http://www.r-project.org.

[46] Paradis E, Claude J, Strimmer K. APE: Analyses of Phylogenetics and Evolution in R language. Bioinformatics. 2004;20: 289–290. doi: 10.1093/bioinformatics/btg412 14734327

[47] Robinson MD, McCarthy DJ, Smyth GK. edgeR: a Bioconductor package for differential expression analysis of digital gene expression data. Bioinformatics. 2010;26: 139–140. doi: 10.1093/bioinformatics/btp616 19910308 PMC2796818

[48] Benjamini Y, Hochberg Y. Controlling the false discovery rate: A practical and powerful approach to multiple testing. J R Stat Soc. 1995;57: 289–300.

[49] Langfelder P, Horvath S. WGCNA: an R package for weighted correlation network analysis. BMC Bioinformatics. 2008;9: 559. doi: 10.1186/1471-2105-9-559 19114008 PMC2631488

[50] Spelt C, Quattrocchio F, Mol JNM, Koes R. Anthocyanin1 of petunia encodes a basic helix-loop-helix protein that directly activates transcription of structural anthocyanin genes. Plant Cell. 2000;12: 1619–1631. doi: 10.1105/tpc.12.9.1619 11006336 PMC149074

[51] Gopaulchan D, Umaharan P, Lennon AM. A molecular assessment of the genetic model of spathe color inheritance in *Anthurium andraeanum* (Hort.). Planta. 2014;239: 695–705. doi: 10.1007/s00425-013-2007-9 24363030

[52] Gopaulchan D, Lennon AM, Umaharan P. Expression analysis of the anthocyanin genes in pink spathes of anthurium with different color intensities. J Am Soc Hortic Sci. 2015;140: 480–489. doi: 10.21273/jashs.140.5.480

[53] Zhang C, Yao X, Ren H, Wang K, Chang J. Isolation and characterization of three Chalcone synthase genes in pecan (*Carya illinoinensis*). Biomolecules. 2019;9. doi: 10.3390/biom9060236 31216753 PMC6627513

[54] Deng X, Bashandy H, Ainasoja M, Kontturi J, Pietiäinen M, Laitinen RAE, et al. Functional diversification of duplicated chalcone synthase genes in anthocyanin biosynthesis of Gerbera hybrida. New Phytol. 2014;201: 1469–1483. doi: 10.1111/nph.12610 24266452

[55] Sun W, Meng X, Liang L, Jiang W, Huang Y, He J, et al. Molecular and Biochemical Analysis of Chalcone Synthase from Freesia hybrid in Flavonoid Biosynthetic Pathway. PLoS One. 2015;10: e0119054. Available: doi: 10.1371/journal.pone.0119054 25742495 PMC4351062

[56] Zhou L, Wang Y, Ren L, Shi Q, Zheng B, Miao K, et al. Overexpression of Ps-CHI1, a homologue of the chalcone isomerase gene from tree peony (*Paeonia suffruticosa*), reduces the intensity of flower pigmentation in transgenic tobacco. Plant Cell, Tissue Organ Cult. 2014;116: 285–295. doi: 10.1007/s11240-013-0403-2

[57] Lim W, Li J. Co-expression of onion chalcone isomerase in Del/Ros1-expressing tomato enhances anthocyanin and flavonol production. Plant Cell, Tissue Organ Cult. 2017;128: 113–124. doi: 10.1007/s11240-016-1090-6

[58] Ravaglia D, Espley R V, Henry-Kirk RA, Andreotti C, Ziosi V, Hellens RP, et al. Transcriptional regulation of flavonoid biosynthesis in nectarine (*Prunus persica*) by a set of R2R3 MYB transcription factors. BMC Plant Biol. 2013;13: 68. doi: 10.1186/1471-2229-13-68 23617716 PMC3648406

[59] Peng Y, Lin-Wang K, Cooney JM, Wang T, Espley R V., Allan AC. Differential regulation of the anthocyanin profile in purple kiwifruit (Actinidia species). Hortic Res. 2019;6. doi: 10.1038/s41438-018-0076-4 30622721 PMC6312553

[60] Sun W, Shen H, Xu H, Tang X, Tang M, Ju Z, et al. Chalcone Isomerase a Key Enzyme for Anthocyanin Biosynthesis in *Ophiorrhiza japonica*. Front Plant Sci. 2019;10: 865. doi: 10.3389/fpls.2019.00865 31338101 PMC6629912

[61] Pillet J, Yu H-W, Chambers AH, Whitaker VM, Folta KM. Identification of candidate flavonoid pathway genes using transcriptome correlation network analysis in ripe strawberry (Fragaria × ananassa) fruits. J Exp Bot. 2015/05/15. 2015;66: 4455–4467. doi: 10.1093/jxb/erv205 25979996 PMC4507756

[62] Luo P, Ning G, Wang Z, Shen Y, Jin H, Li P, et al. Disequilibrium of Flavonol Synthase and Dihydroflavonol-4-Reductase Expression Associated Tightly to White vs. Red Color Flower Formation in Plants. Front Plant Sci. 2016;6: 1257. doi: 10.3389/fpls.2015.01257 26793227 PMC4710699

[63] Tian J, Han Z, Zhang J, Hu Y, Song T, Yao Y. The Balance of Expression of Dihydroflavonol 4-reductase and Flavonol Synthase Regulates Flavonoid Biosynthesis and Red Foliage Coloration in Crabapples. Sci Rep. 2015;5: 12228. doi: 10.1038/srep12228 26192267 PMC4507444

[64] Walker AR, Lee E, Bogs J, McDavid DAJ, Thomas MR, Robinson SP. White grapes arose through the mutation of two similar and adjacent regulatory genes. Plant J. 2007;49: 772–785. doi: 10.1111/j.1365-313X.2006.02997.x 17316172

[65] Qian M, Yu B, Li X, Sun Y, Zhang D, Teng Y. Isolation and Expression Analysis of Anthocyanin Biosynthesis Genes from the Red Chinese Sand Pear, *Pyrus pyrifolia Nakai* cv. *Mantianhong*, in Response to Methyl Jasmonate Treatment and UV-B/VIS Conditions. Plant Mol Biol Report. 2014;32: 428–437. doi: 10.1007/s11105-013-0652-6

[66] Zhang B, Horvath S. A General Framework for Weighted Gene Co-Expression Network Analysis. Stat Appl Genet Mol Biol. 2005;4. doi: 10.2202/1544-6115.1128 16646834

[67] Xi W, Feng J, Liu Y, Zhang S, Zhao G. The R2R3-MYB transcription factor PaMYB10 is involved in anthocyanin biosynthesis in apricots and determines red blushed skin. BMC Plant Biol. 2019;19: 287. doi: 10.1186/s12870-019-1898-4 31262258 PMC6604168

[68] Song T, Li K, Wu T, Wang Y, Zhang X, Xu X, et al. Identification of new regulators through transcriptome analysis that regulate anthocyanin biosynthesis in apple leaves at low temperatures. PLoS One. 2019;14: e0210672. Available: doi: 10.1371/journal.pone.0210672 30695036 PMC6350969

[69] He Y, Wang Z, Ge H, Liu Y, Chen H. Weighted gene co-expression network analysis identifies genes related to anthocyanin biosynthesis and functional verification of hub gene SmWRKY44. Plant Sci. 2021;309: 110935. doi: 10.1016/j.plantsci.2021.110935 34134842

[70] Liu Y, Zhou B, Qi Y, Chen X, Liu C, Liu Z, et al. Expression Differences of Pigment Structural Genes and Transcription Factors Explain Flesh Coloration in Three Contrasting Kiwifruit Cultivars. Front Plant Sci. 2017;8: 1507. doi: 10.3389/fpls.2017.01507 28919902 PMC5586210

[71] Ortiz-Barrientos D. The color genes of speciation in plants. Genetics. 2013;194: 39–42. doi: 10.1534/genetics.113.150466 23633142 PMC3632479

[72] Ohmiya A. Molecular mechanisms underlying the diverse array of petal colors in chrysanthemum flowers. Breed Sci. 2018/02/17. 2018;68: 119–127. doi: 10.1270/jsbbs.17075 29681754 PMC5903973

[73] Gao L, Yang H, Liu H, Yang J, Hu Y. Extensive transcriptome changes underlying the flower color intensity variation in *Paeonia ostii*. Front Plant Sci. 2016;6: 1–16. doi: 10.3389/fpls.2015.01205 26779235 PMC4702479

[74] Quattrocchio F, Wing JF, Leppen HTC, Mol JNM, Koes RE. Regulatory genes controlling anthocyanin pigmentation are functionally conserved among plant species and have distinct sets of target genes. Plant Cell. 1993;5: 1497–1512. doi: 10.1105/tpc.5.11.1497 12271045 PMC160381

[75] de Vetten N, Quattrocchio F, Mol J, Koes R. The an11 locus controlling flower pigmentation in petunia encodes a novel WD-repeat protein conserved in yeast, plants, and animals. Genes Dev. 1997;11: 1422–1434. doi: 10.1101/gad.11.11.1422 9192870

[76] Quattrocchio F, Wing J, van der Woude K, Souer E, de Vetten N, Mol J, et al. Molecular analysis of the anthocyanin2 gene of petunia and its role in the evolution of flower color. Plant Cell. 1999;11: 1433–1444. doi: 10.1105/tpc.11.8.1433 10449578 PMC144295

