# Supplementary figures and images for: Comparative transcriptomic analysis reveals key components controlling spathe color in Anthurium andraeanum (Hort.)
Source: PLoS One. 2021 Dec 10;16(12):e0261364. doi: 10.1371/journal.pone.0261364 (PMC8664202; doi:10.1371/journal.pone.0261364)

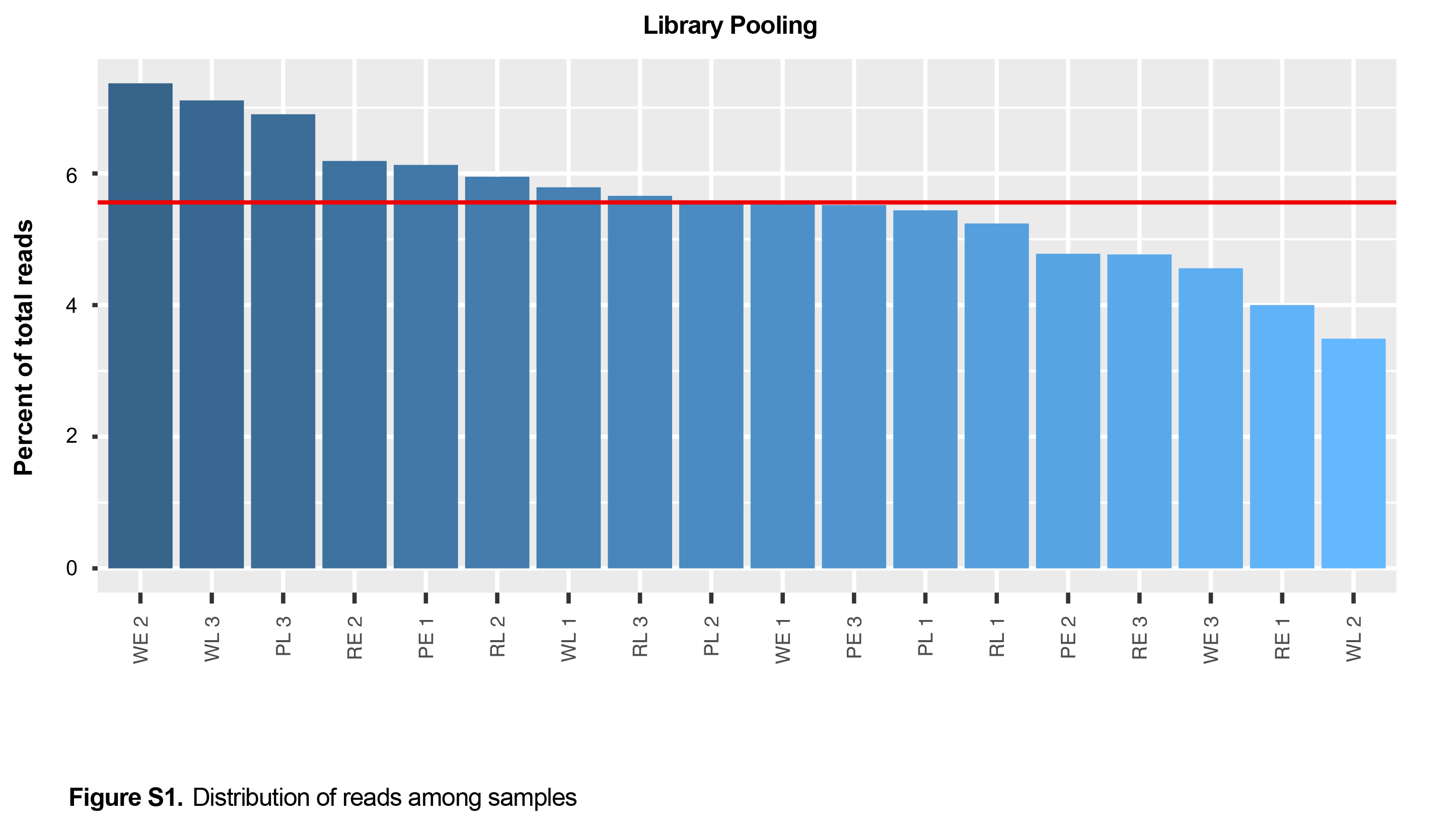

Supplement: S1 Fig — (TIF) [file pone.0261364.s001.tif]

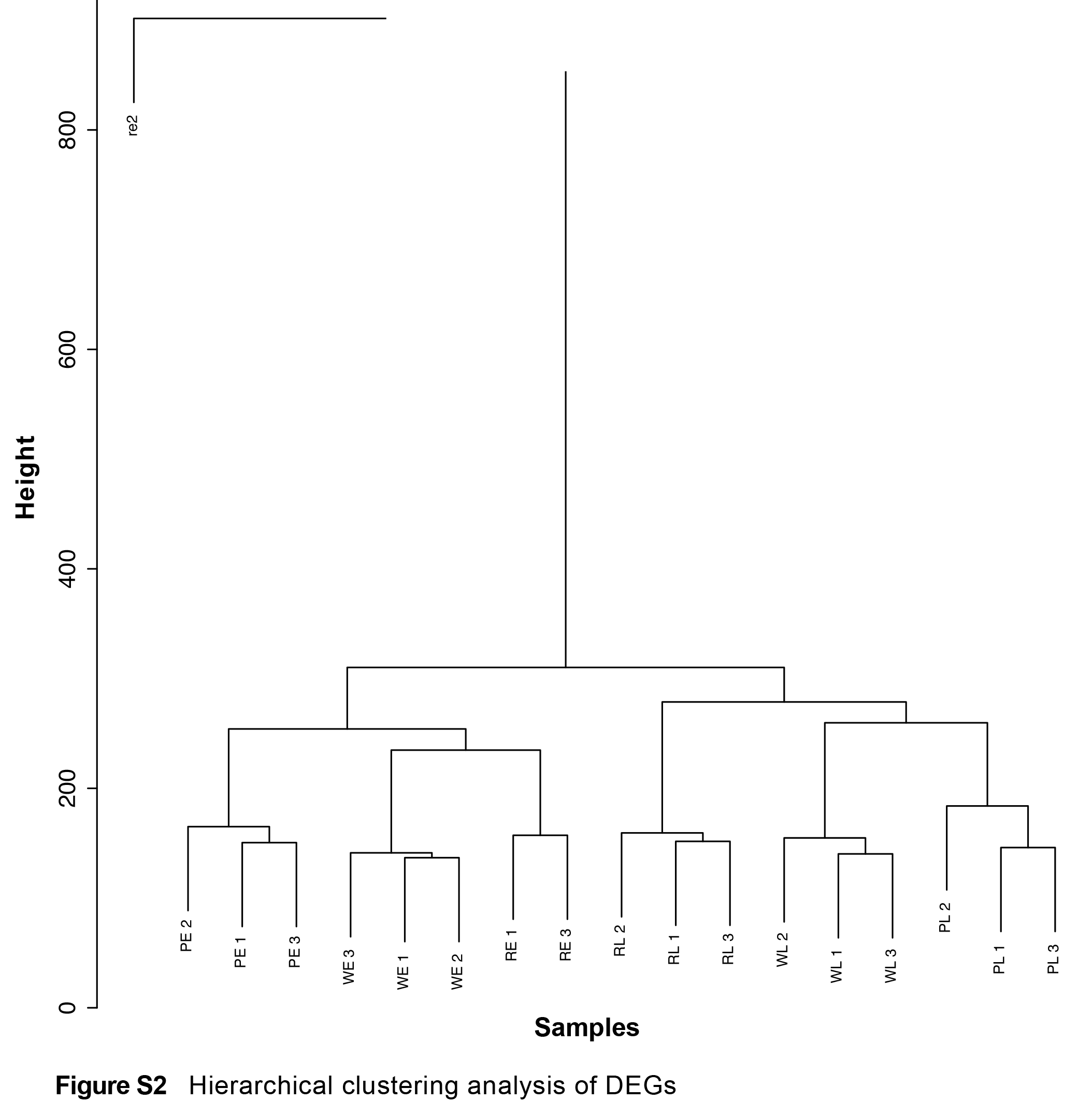

Supplement: S2 Fig — (TIF) [file pone.0261364.s002.tif]
